# Cellular transcriptional alterations of peripheral blood in Alzheimer’s disease

**DOI:** 10.1186/s12916-022-02472-4

**Published:** 2022-08-29

**Authors:** Liting Song, Yucheng T. Yang, Qihao Guo, the ZIB Consortium, Xing-Ming Zhao

**Affiliations:** 1 Institute of Science and Technology for Brain-Inspired Intelligence, Fudan University, Shanghai, 200433 China; 2 MOE Key Laboratory of Computational Neuroscience and Brain-Inspired Intelligence, and MOE Frontiers Center for Brain Science, Fudan University, Shanghai, 200433 China; 3 Zhangjiang Fudan International Innovation Center, Shanghai, 200433 China; 4 Department of Gerontology, Shanghai Jiao Tong University Affiliated Sixth People’s Hospital, Shanghai, 200233 China; 5 International Human Phenome Institutes (Shanghai), Shanghai, 200433 China

**Keywords:** Alzheimer’s disease (AD), Mild cognitive impairment (MCI), Peripheral blood transcriptome, Deconvolution, Cell-specific gene expression, Cellular proportion

## Abstract

**Background:**

Alzheimer’s disease (AD), a progressive neurodegenerative disease, is the most common cause of dementia worldwide. Accumulating data support the contributions of the peripheral immune system in AD pathogenesis. However, there is a lack of comprehensive understanding about the molecular characteristics of peripheral immune cells in AD.

**Methods:**

To explore the alterations of cellular composition and the alterations of intrinsic expression of individual cell types in peripheral blood, we performed cellular deconvolution in a large-scale bulk blood expression cohort and identified cell-intrinsic differentially expressed genes in individual cell types with adjusting for cellular proportion.

**Results:**

We detected a significant increase and decrease in the proportion of neutrophils and B lymphocytes in AD blood, respectively, which had a robust replicability across other three AD cohorts, as well as using alternative algorithms. The differentially expressed genes in AD neutrophils were enriched for some AD-associated pathways, such as ATP metabolic process and mitochondrion organization. We also found a significant enrichment of protein-protein interaction network modules of leukocyte cell-cell activation, mitochondrion organization, and cytokine-mediated signaling pathway in neutrophils for AD risk genes including *CD33* and *IL1B*. Both changes in cellular composition and expression levels of specific genes were significantly associated with the clinical and pathological alterations. A similar pattern of perturbations on the cellular proportion and gene expression levels of neutrophils could be also observed in mild cognitive impairment (MCI). Moreover, we noticed an elevation of neutrophil abundance in the AD brains.

**Conclusions:**

We revealed the landscape of molecular perturbations at the cellular level for AD. These alterations highlight the putative roles of neutrophils in AD pathobiology.

**Supplementary Information:**

The online version contains supplementary material available at 10.1186/s12916-022-02472-4.

## Background

Alzheimer’s disease (AD) is a progressive neurodegenerative disorder that is characterized by cognitive and functional impairment and memory loss [1, 2]. It is the most common frequent form of dementia leading to a wide spectrum of social and financial burdens [3]. Therefore, there is an urgent need to understand the etiology and pathogenesis of the disease. For decades, the amyloid cascade hypothesis was the major pathogenic theory. The aberrant processing of Amyloid-beta precursor protein or dysfunctional clearance of the amyloid-β peptide (Aβ) leads to the accumulation of the Aβ plaque, followed by the deposition of neurofibrillary tangles (NFTs), and ultimately causes synaptic and neuronal dysfunction and loss [4]. However, the failure of multiple therapeutic trials targeting Aβ clearance suggests that there must be other pathogenic mechanisms, such as inflammation [5, 6].

Neuroinflammation is a dominant manifestation and a contributor to AD pathogenesis [7]. Initially, microglia and astrocytes are activated by the deposition of Aβ plaques and other pathological proteins to phagocytose Aβ peptides, thus reducing the plaque burden and providing a neuroprotective function [8–11]. However, the persistence of plaque results in the constant production of inflammatory cytokines, chemokines, and neurotoxins by these cells, which ultimately impairs both neurons and other cells (astrocytes, oligodendrocytes, and microglia) [12–14]. The vicious circle finally causes neurodegeneration and neuron loss. Recently, some genome-wide association studies (GWAS) have found the association between AD and key microglial genes, including *CD33* and *TREM2* genes involved in innate immunity [15–18], suggesting immune system-mediated actions contribute to or drive AD pathogenesis.

Epidemiological and translational studies have indicated that the peripheral systemic immune or inflammatory responses may promote neurodegenerative and AD-specific pathology [19]. For instance, systemic immune challenge by the lipopolysaccharide and chronic inflammatory disorders could result in the development of AD-like neuropathology and an increase in the risk of AD [20–23], respectively. Individuals with higher levels of systemic inflammation in the blood during midlife exhibit a steeper cognitive decline [24]. Accordingly, non-steroidal anti-inflammatory drugs could reduce the risk of AD [25, 26]. These findings highlight the involvement of systemic peripheral inflammation in AD etiology and progression.

Emerging evidence support that there is an intense crosstalk between the peripheral systemic immune and the central nervous systems [27]. For instance, cytokines such as interleukin-6 (IL-6), IL-1β, and tumor necrosis factor (TNF) in the blood could signal to brain via the circumventricular organs and transport across the blood-brain barrier (BBB) via receptors on the endothelium, leading to increased activation of microglia [27–29]. Furthermore, the infiltration of innate and adaptive immune cells from peripheral blood, containing monocytes, macrophages, neutrophils, and lymphocytes, also have been observed in the brains of AD patients and animal models [9, 30–32]. With the stimulation of Aβ and the neuroinflammation, monocytes migrate to brain parenchyma and change their expression profiles and morphology termed bone marrow-derived microglia to phagocytose Aβ [33, 34]. Neutrophils enter the brain via LFA-1 integrin and surround Aβ plaques with neutrophil extracellular traps, potentially promoting BBB damage and neuronal toxicity [35, 36]. Activated CD8^+^ T cells exhibit a higher proportion in the CSF of AD patients than healthy older adults and correlate with clinical and structural markers of AD pathology [37]. Several studies based on the single-cell RNA sequencing of peripheral blood mononuclear cells further have disclosed the cell-specific signatures in AD and mild cognitive impairment (MCI) [38–40]. However, the small sample size and underestimate of polymorphonuclear leukocytes (e.g., neutrophils) limit our understanding of the distribution and biological characteristics of all the peripheral blood immune cells.

Here, to explore the alterations of cellular composition and changes of intrinsic expression of blood immune cell types in AD, we performed cellular deconvolution in large-scale bulk blood transcriptomic cohorts and evaluated the association between the cellular proportion and disease status and cognitive functions. Moreover, we identified shared and specific biological processes among cell types. Then, we performed enrichment analysis for AD risk genes on network modules of neutrophils. Finally, we explored the infiltration of immune cells in AD brain regions. These cellular proportion and signaling perturbations contribute to further research regarding disease etiology and the development of immune-related diagnostic and/or therapeutic biomarkers.

## Methods

### Datasets

Blood expression data was obtained from the Alzheimer’s Disease Neuroimaging Initiative (ADNI) database [41, 42], a longitudinal multicenter dataset in the US and Canada containing clinical, imaging, genetic, and biochemical data to identify biomarkers for early detection and progression of AD. Enrollment and diagnosis criteria for ADNI have been described previously in detail [43]. Briefly, enrolled participants were between 55 and 90 years old without other psychiatric and neurologic diseases. Subjects with AD were diagnosed with the National Institute of Neurological and Communicative Disorders and Stroke-Alzheimer’s Disease and Related Disorders Association (NINCDS-ADRDA) criteria for probable AD [44]. Subjects with MCI and normal control (NC) participants were determined through whether meeting the Jak/Bondi Criteria or not with neuropsychological measures [45]. The expression data were sequenced using the Affymetrix Human Genome U 219 array and normalized by the Robust Multichip Analysis (RMA) [46].

To validate the findings in ADNI, we performed the same analysis on other three independent peripheral blood transcriptomic datasets, including the AddNeuroMed datasets ANM1 [47] and ANM2 [47], and the AD cohort in Zhangjiang international Brain Biobank (ZIB-AD [48]). The ANM consortium is a large cross-European, public-private consortium for AD [49]. Blood RNA samples from ANM1 and ANM2 were profiled on Illumina Human HT-12 v3 and v4 Expression BeadChips, respectively. The probe-set level intensities of each set were normalized using the RMA method in the R package affy [50]. The ZIB is an ongoing imaging genetic neuropsychiatric cohort in China. Expression profiling of AD cohort in the ZIB was sequenced on Illumina NovaSeq^TM^ 6000 with 150-bp paired-end. In addition, we also performed cellular deconvolution on a human AD brain transcriptomic dataset (i.e., Mayo RNAseq [51, 52]) and a spatial transcriptomics dataset in brain of AD mouse model (i.e., Alzmap [53, 54]).

### Estimation of cellular proportion of immune cell types

We first applied EPIC [55] to estimate the cellular proportions based on deconvolution algorithm. Briefly, the bulk gene expression (*b*) is modeled as the sum of the expression from the pure cell types composing this sample, i.e., *b* = *C × p*, *C* is a reference gene expression matrix for cell types; and *p* is a vector of the proportions from the *K* cell types. The reference cell gene expression profile was obtained from RNA-Seq data of sorted immune cells [56–58]. The cellular proportions were then inferred by a least-square optimization. EPIC estimates showed a remarkable agreement with the cell fractions computed with flow cytometry in both blood and tumor samples [55].

To avoid the method bias, we also quantified the immune cells with other computational tools, including quanTIseq [59], CIBERSORT [60], and MCP-counter [61]. QuanTIseq is based on a signature matrix derived from 51 RNA-seq data sets of blood-derived immune cells from ten different immune cell types. Both EPIC and QuanTIseq allow estimating the fraction of uncharacterized cells. CIBERSORT, demonstrating robust deconvolution performance and high accuracy in microarray data via nu support vector regression [60], was also applied to estimate the cell fractions and to infer sample-level gene expression for each cell type. A leukocyte RNA-Seq signature matrix comprised of six peripheral blood immune subsets (GSE60424) was taken as the specialized knowledgebase [57, 60]. The above three methods force the estimates to be non-negative and to sum up to one and therefore could be interpreted directly as cell fractions and compared across different immune cell types. Furthermore, MCP-counter, a transcriptomic marker-based approach [61], was applied to quantify the absolute abundance of immune cells. Here, we applied the R package immunedeconv [62] to implement uniformly the above algorithms with a non-log scale gene expression matrix as the input.

### Identification of bulk and cell-type-specific differentially expressed genes

Limma [63], an R package based on linear regression for both RNA-seq and microarray data, was applied to identify bulk differentially expressed genes (DEGs, *P* < 0.01) of AD and MCI in each dataset. The normalized expression matrixes of microarray and RNA-seq data were used as inputs while adjusting for covariates of age and gender. In addition, the Wilcoxon test that performs robustly with a lower false discovery in population studies [64] was applied to further validate the DEGs.

TOAST [65], a reference-free deconvolution method, was applied to detect differentially expressed genes in each cell type between AD or MCI vs. NC. First, we made a model design using a phenotype matrix (including age, gender, and disease status) and the cellular proportions estimated from EPIC. Then, we fitted the linear model for non-log-transformed expression data and the model design. Finally, we tested the disease effects (i.e., differences) in each cell type while controlling for covariates.

### Pathway enrichment analysis

We identified the enriched biological processes in Gene Ontology for DEGs using Metascape [66, 67], which utilizes the hypergeometric test and Benjamini-Hochberg *p*-value correction algorithm to identify significant terms. To address the issue of term redundancies, Metascape will compute pairwise similarity scores between any two enriched terms based on a Kappa-test score [68] and performs hierarchical clustering using the similarity matrix. Then, the hierarchical tree is trimmed with the 0.3 similarity threshold into separate clusters. Metascape choose the most significant (lowest *P*-value) term within each cluster to represent the cluster in the bar graph and heatmap representations. We also constructed enrichment networks, in which the nodes represent significant terms and the edges connect these nodes for which the similarity scores are above 0.3. The networks were visualized using Cytoscape v3.8.0 [69].

### Re-construction of protein-protein interaction networks

For DEGs in neutrophils, we re-constructed protein-protein interaction (PPI) network using Metascape under the combined (all) option. Metascape extracts protein interactions from STRING [70], OmniPath [71], InWeb_IM [72], and BioGrid [73] database for input gene/proteins. Then, the Molecular Complex Detection (MCODE), a graph-theoretic clustering algorithm based on vertex weighting by local neighborhood density and outward traversal from a locally dense seed protein to isolate the dense regions, was applied to identify densely-connected modular clusters with default parameters [74]. For each cluster, Metascape further applies function enrichment analysis and uses the top enriched terms to annotate its biological roles. We also identified candidate hub genes with high degree centralites or clustering coefficients using cytoHubba.

### Statistical analysis

All statistical analyses were performed using R (version 4.0.2). ANOVA (analysis of variance) test and chi-squared test were applied to compare continuous demographics variables and categorical variables, respectively. Comparison of cell proportion between groups was performed using the two-tailed Wilcoxon test, whereas the one-tailed Wilcoxon test was applied to determine whether the NLR of AD and MCI is higher than NC or not. Correlations between proportion and phenotype traits were assessed with Spearman’s correlation. Overlaps of DE genes between two sets were assessed using a hypergeometric test. The enrichment of PPI modules in AD risk/associated gene lists was determined with the application of Fisher’s exact test.

## Results

### Alterations of immune cell proportion in AD

**Fig. 1:**
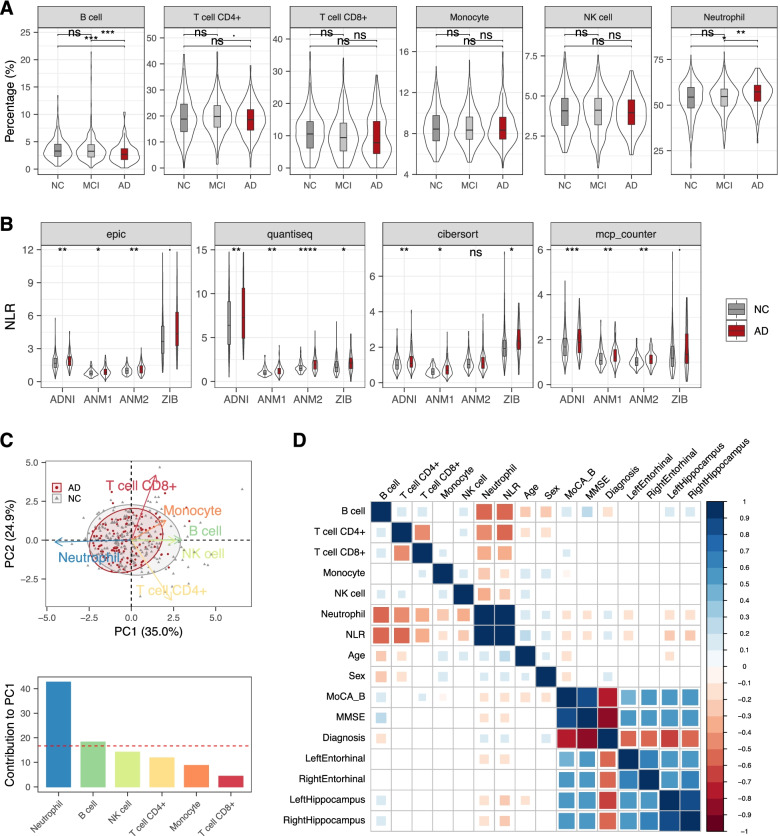
Peripheral immune cell proportion in AD. **A** The distribution of cellular proportion of six peripheral blood immune cells in MCI, AD individuals, and controls. Boxplot shows the median and interquartile range, with the outer violin plot showing the full distribution of data. ns, not significant; •, *P* < 0.1; *, *P* ≤ 0.05; **, *P* ≤ 0.01; ***, *P* ≤ 0.001; Wilcoxon test. **B** Comparison of the neutrophil-to-lymphocyte ratio (NLR) between AD and NC across different datasets under different deconvolution tools. The one-tailed Wilcoxon test was applied to test whether NLR in AD was higher than in NC. **C** Principal component analysis (PCA) of cellular proportions in AD and NC participants. Each point represents one participant’s scores on the first 2 principal components (PC1 and PC2), where grey and red points correspond to NC and AD, respectively. The blue arrows show the loadings of cell types on the first 2 principal components. Ellipses show the 80% confidence ellipse for each group. The bottom bar plot shows the contribution of each cell type to PC1. The dashed red line represents the expected value if the contribution were uniform. **D** Spearman correlations between immune cell proportion, diagnosis, cognitive measurements, brain-area volumes, and demographic variables. Only significant correlations with *P* < 0.05 are shown. Color represents the correlation coefficient. Blue and red colors indicate positive and negative correlations, respectively. MoCA-B, Montreal Cognitive Assessment Basic; MMSE, Mini-Mental State Examination

We obtained the blood-derived gene expression data from the individuals in the ADNI database [41], including 116 AD, 382 MCI, and 246 elderly NC. We first applied a deconvolution algorithm EPIC [55] to estimate the immune cell proportions for these samples using the gene expression profiles of the reference immune cell types [56–58] (Fig. 1A). We found that the proportion of B cells was significantly lower in AD compared to NC (*P* = 3.3e−4, Wilcoxon test) and MCI (*P* = 9.7e−4, Wilcoxon test), while the proportion of neutrophil was significantly higher in AD compared to NC (*P* = 4.8e−3, Wilcoxon test). Then, we used the neutrophil-to-lymphocyte ratio (NLR), a simple ratio between the neutrophil and lymphocyte counts measured in peripheral blood, to estimate the neutrophil-mediated systemic inflammation for these samples. We found that the NLR in AD exhibited a significant increase compared to controls (*P* = 9.9e-3, one-tailed Wilcoxon test) (Fig. 1B). To further validate this observation, we also estimated the immune cell proportions in other three independent bulk blood transcriptomic datasets (i.e., ANM1, ANM2 [47], and ZIB [75]) using three alternative computational tools (i.e., quanTIseq [59], CIBERSORT [60], and MCP-counter [61]). As expected, we found a significantly higher NLR in AD than in NC in most of the datasets when using different computational tools except for ANM2 dataset under CIBERSORT (Fig. 1B). Although the NLR difference between MCI and NC is not statistically significant in most tests (Additional file 1: Fig. S1), we could still observe the same trend as in AD vs. NC. Collectively, these findings revealed an elevation of inflammation response in AD and MCI than in NC.

We then performed principal component analysis (PCA) on the immune cell proportion from these samples. The first two principal components (PCs) can explain 59.9% of the total variance-covariance of the immune cell proportion in the samples. The PC1 (35% of the total variance-covariance) was most strongly contributed by neutrophils (negative) and B cells (positive) (Fig. 1C). The PC2 (24.9% of the total variance-covariance) was most strongly weighted on CD8^+^ and CD4^+^ T cells. The PCA scatterplot revealed that the immune cell profiles in most AD cases could not be distinguished from those in controls (Fig. 1C), but a subset of them showed lower PC1 positivity scores, i.e., a distinctively lower proportion of B cells and/or an increased proportion of neutrophils, indicating a lower adaptive activity and/or a higher innate and pro-inflammatory activity in these samples.

We then estimated the correlations between immune cell proportion, cognition, disease diagnosis, brain structural features for early detection of AD [76] (derived from MRI data), and demographic variables in NC and AD participants (Fig. 1D). Consistent with the above results, NLR and neutrophil proportion showed positive correlation with disease diagnosis, while exhibited negative correlations with hippocampus and left entorhinal cortex volumes, as well as with cognitive functions (Fig. 1D and Additional file 1: Fig. S2). A similar pattern of correlation could be observed when estimating using all AD, MCI and NC individuals (Additional file 1: Fig. S3). Moreover, we noticed that the cellular proportion and NLR could be affected by age and gender (Fig. 1D). Considering the variation of age and gender across different diagnosis groups (Additional file 1: Fig. S4) that could be important confounding factors, we therefore performed logistic regression between disease status and cellular proportion by adjusting for the two confounding factors (i.e. age and gender) (Additional file 2: Table S1). After adjusting for the confounding factors, although with a decreased statistical significance level, the proportions of neutrophils and B cells remained to show significantly positive (*t* = 2.112, *P* = 0.035, logistic regression) and negative (*t* = − 2.553, *P* = 0.0111, logistic regression) correlation with AD status in ADNI dataset, respectively. We also confirmed these results in ANM1 and ANM2 datasets (Additional file 2: Table S1). We observed the same-direction while insignificant correlation between the proportions of neutrophils and B cells and MCI status (Additional file 2: Table S1). In addition, the difference of the proportion of CD4^+^ T cells between MCI and AD did not remain statistically significant (*P* = 0.262, logistic regression) after adjusting for the confounding factors of age and gender. Consistent with previous studies [77], these results indicated that in addition to age and gender factors, the occurrence of AD partially drove the increase in neutrophil proportion and NLR.

### Cell-type-aware DEGs in AD

**Fig. 2:**
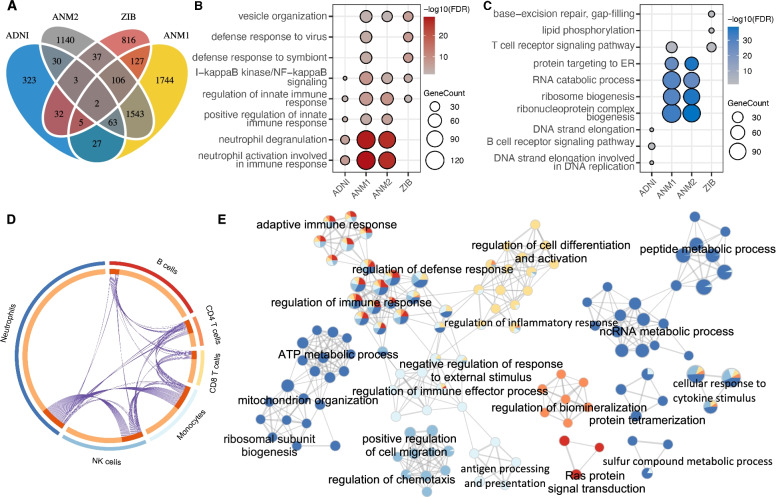
Differentially expressed genes (DEG) in AD at bulk and cellular levels. **A** The Venn diagram shows the overlap of bulk DEGs among four blood transcriptomic datasets. **B**, **C** The top enriched biological processes of upregulated (**B**) and downregulated (**C**) genes in AD compared with NC in each dataset. The point size represents the number of DE genes in the corresponding pathway. Color represents the significant level (i.e., FDR). **D** Circos plot shows the overlap between DE genes in each cell type in ADNI, where colors of the outer circle represent cell types. The dark and light oranges in the inner circle indicate genes hitting multiple cell types and only one cell type, respectively. Purple curves link identical genes. **E** Network of enriched terms in each cell type in ADNI, where terms with a similarity score > 0.3 are connected by edges. The color of the pie chart indicates the cell type which is color-coded as Fig. 2D, where the size of a slice represents the percentage of DEG from each cell type. The node size represents the number of DEG hitting terms

The differentially expressed genes (DEGs) in AD were identified in each dataset through linear regression by adjusting for the co-founding factors (Fig. 2A), where the significant overlap and strong concordance with those identified using the Wilcoxon test confirmed the reliability of the results (Additional file 1: Fig. S5). Consistent with the changes of immune cell proportion and previous studies [78], we found that the bulk upregulated and downregulated genes in AD were significantly enriched in neutrophil activation/degranulation and B/T cell receptor signaling pathways in three out of four datasets, respectively (Fig. 2B, C). We also observed downregulation of neutrophil activation/degranulation and upregulation of RNA catabolic process in MCI in ANM1 and ANM2 datasets (Additional file 1: Fig. S6).

To further examine the intrinsic expression changes of individual immune cell types, we identified the DEGs in each cell type by adjusting for the cell proportion and co-founding factors in ADNI dataset (Fig. 2D). We compared the bulk DEGs and cell-type-intrinsic DEGs (Additional file 1: Fig. S7) and found that only a small fraction of the DEGs in each cell type overlap with the bulk DEGs. We noticed that four (i.e., neutrophils, monocytes, NK cells and CD4^+^ T cells) out of the six cell types exhibited significant overlap with the bulk DEGs; the neutrophils showed the strongest overlap *(P* = 9.5e−11, hypergeometric test), which may be due to the high abundance (55-70%) of this cell type in the peripheral blood.

We next explored the shareness of the perturbed biological pathways across the peripheral immune cell types in AD from ADNI dataset. As shown in Fig. 2E, the dysregulated genes in all or most of the cell types are associated with the regulation of immune response, regulation of defense response, cellular response to cytokine stimulus, and regulation of inflammatory response, which is consistent with the common dysregulated signalings among peripheral blood mononuclear cells in AD revealed by single-cell RNA-seq analysis [40]. Moreover, we observed cell-type-specific disruptions in some biological processes, for instance, Ras protein signal transduction in B cells, positive regulation of cell migration in NK cells, regulation of biomineralization in CD4^+^ T cells, antigen processing, and presentation in monocytes. Interestingly, we found that there was a significant enrichment of DEGs in neutrophils for ncRNA metabolic process, peptide and ATP metabolic process, ribosomal subunit biogenesis, and mitochondrion organization, which were also downregulated in the bulk level, indicating that the neutrophils could be the primary factor in the peripheral transcriptomic alterations. The mitochondrion dysfunction and energy hypometabolism have been reported as one of the most consistent and earliest abnormalities in AD and MCI and could be a link to Aβ production [79], highlighting the putative roles of neutrophils in the pathobiology of AD.

### Enrichment of AD risk genes in neutrophil modules

**Fig. 3:**
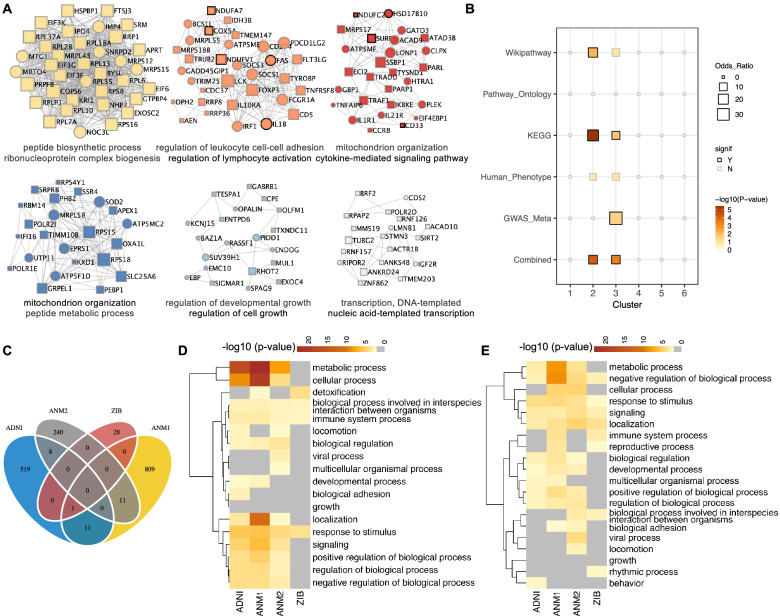
Protein-protein interaction network identified in neutrophils. **A** Protein-protein interaction network and top MCODE module clusters for DEGs in neutrophils of AD. The bottom shows the top enriched biological processes for each module. Circle and square indicate the up and downregulated genes of neutrophils in AD compared with NC. Node size represents the degree. Genes associated with AD are highlighted with a black border. **B** The enrichment of each module cluster in AD-associated gene lists, where the black border represents significant enrichment. Color and size of node indicate the significance and odds ratio of enrichment, respectively. **C** The Venn diagram shows the overlap of DEGs in neutrophils of AD versus NC across four blood transcriptomic datasets. **D**, **E** Heatmap of top enriched biological process terms’ parents of DEGs in AD (D) and MCI (E) across four datasets, colored by *P*-values

The protein-protein interaction (PPI) networks can help elucidate the biochemical complexes or signal transduction components that govern the biological outputs. Given the putative functions of neutrophils in AD pathogenesis, we re-constructed PPI networks for the DEGs in neutrophils using Metascape [66, 67]. We identified densely connected modular clusters (with a minimum size of three proteins) which represent specific molecular complexes and functional units [80, 81] (Fig. 3A and Additional file 2: Table S2). For example, the most significant modular cluster #1 is enriched in ribonucleoprotein complex biogenesis, including ribosome protein families, such as *RPL35*, *RPL7A*, and *RPL6*. The modular clusters #2 and #3 are enriched in cytokine-mediated signaling, including interleukins (ILs) and tumor necrosis factors (TNFs). We also observed other biologically meaningful functional units enriched in clusters #3, #4, and #5, including ATP metabolic process, mitochondrial transport, and mitochondrial outer membrane-associated with mitochondrial organization.

To confirm that the modular clusters are associated with AD pathogenesis, we then estimated the enrichment of the genes in the modular clusters in the AD genetic risk gene set or the AD-associated gene sets from diverse sources (Fig. 3B). We found that modular clusters #2 and #3 were significantly enriched in more than one AD-related gene sets. The modular cluster #2 exhibited mainly enrichment in the regulation of leukocyte cell-cell adhesion and activation. Interestingly, we found that two hub genes in modular cluster #2, *IL1B* (encoding pro-inflammation members of the interleukin 1 cytokine family) and *FAS* (encoding TNF-receptor superfamily), were upregulated in neutrophils from AD participants (Fig. 3B, Additional file 1: Fig. S8A-B). We also noticed that modular cluster #3, a mitochondrion organization and cytokine-mediated signaling pathway-related module, was significantly enriched for AD genetic risk genes from GWAS and meta-analysis of large LOAD consortium data sets [82]. For example, the expression level of *CD33* (encoding a member of the sialic acid-binding Ig-like lectin family of receptors and expressed on myeloid cells and microglia [83, 84]) shows positive correlation with the hippocampus and entorhinal cortex volumes (Additional file 1: Fig. S9). In previous studies, it has been reported that *CD33* might play an important role in Aβ clearance and neuroinflammatory pathways mediated by microglia in the brain, and its structural variants and SNP were associated with a higher risk of AD [15, 85]. In addition, we would like to notice that *CD33* was one of the hub genes with high clustering coefficients in modular cluster #3 (Additional file 1: Fig. S8D). These results highlight the putative functions of modular cluster #3 in neutrophils in AD pathogenesis.

We then performed biological process enrichment analysis for the DEGs in neutrophils. We found inhibition of mitochondrion organization and activation of cytokine-mediated signaling pathway and inflammatory response in AD vs. NC (Additional file 1: Fig. S10). To validate the disruptions of signaling pathways in neutrophils, we further performed pathway enrichment analysis using the DEGs identified from different datasets (Fig. 3C-D). Although the DEGs from different datasets exhibited limited overlap (Fig. 3C), we would like to highlight the convergence in the enriched pathway categories across at least three cohorts, including metabolic process, immune system process, and response to stimulus (Fig. 3D, Additional file 2: Table S3). In addition, we observed similar pathway enrichment patterns in MCI across different cohorts (Fig. 3E, Additional file 2: Table S4).

### Elevation of neutrophils in AD brains

Compelling evidence has demonstrated that the dysfunction and deterioration of blood-brain barrier (BBB) may play key roles in AD pathogenesis through a feedback loop with the accumulation of Aβ and accelerate cognitive impairment and the onset of dementia [86–88]. Blood-derived leukocyte subpopulations, such as lymphocytes and monocytes, have been also identified in the brains of AD patients and animal models [6, 30, 89].

**Fig. 4:**
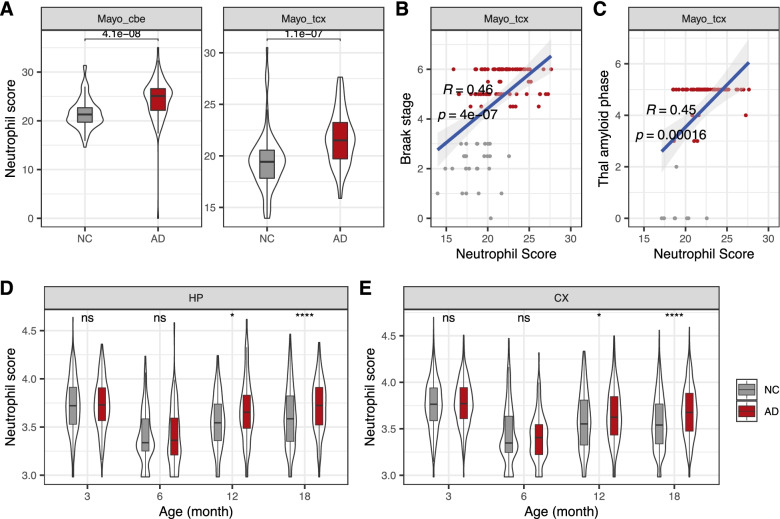
The abundance of neutrophils in AD brain. **A** Comparison of the absolute abundance of neutrophils in the cerebellum (CBE) and temporal cortex (TCX) between AD individuals and controls. **B** Correlation between the neutrophil abundance score and Braak tau neurofibrillary tangle staging score in the temporal cortex. The association was assessed using the Pearson correlation test. **C** Correlation between the neutrophils abundance score and Thal amyloid phasing score. **D**, **E** Comparison of the absolute abundance of neutrophils between AD and NC model mice in the hippocampus (**D**) and brain cortex (**E**) of different ages

To check whether the neutrophils infiltrate into AD brains, we estimated the proportion and abundance of neutrophils in AD brains using transcriptomic dataset from 97 AD individuals and 105 controls (i.e., Mayo RNA-seq [51]). Consistent with the extravasation migration of neutrophils into brain [31, 36], we observed that the absolute abundance of neutrophils was significantly increased in AD brains in both cerebellum (CBE) (*P* = 4.1e−8, Wilcoxon test) and temporal cortex (TCX) (*P* =1.1e−7, Wilcoxon test) (Fig. 4A). We also found that the abundance of neutrophils exhibited significantly positive correlation with Braak tau neurofibrillary tangle staging score (Fig. 4B and Additional file 1: Fig. S11A) and Thal amyloid phase (Fig. 4C and Additional file 1: Fig. S11B) in the two brain regions. However, we did not observe significant difference of the proportions of neutrophils between ADs and NCs (Additional file 1: Fig. S12), which could be due to the massive elevation of brain immune cells (e.g., microglia and astrocytes). Furthermore, we quantified the abundance of neutrophils in a spatial transcriptomics dataset from AD mouse model (i.e., Alzmap in 3, 6, 12, and 18 months old) [53]. As expected, we found that the abundance of neutrophils show significant increase in both hippocampus (HP) and cortex (CX) at the late stages (i.e., 12 and 18 months) instead of at the early stages (3 and 6 months) (Figs. 4D–E).

## Discussion

In this study, we firstly performed deconvolution analysis for a large-scale bulk peripheral blood transcriptome cohort with AD, MCI, and NC participants. We then compared the cellular proportions of immune cells among these groups and further validated the results using independent three blood transcriptomic datasets with alternative computational tools. We next estimated the cell-intrinsical DEGs for the deconvoluted immune cell types. We identified cell-type-specific and shared signaling pathways across these immune cell types. We also re-constructed protein-protein interaction networks for the DEGs in neutrophils and identified several modular clusters that are associated with AD pathogenesis. Finally, we found that the infiltration of immune cells could be elevated in the AD brains.

Broadly, we found that the proportions of neutrophils and B lymphocytes significantly increased and decreased in AD compared to NC, respectively, consistent with an elevation of the NLR in AD. We observed that proportion of neutrophils and the NLR exhibited negative correlations with AD magnetic resonance imaging (MRI) biomarkers, and cognitive measurements, and showed negative correlations with AD status even after adjusting for the confounding factors of age and gender. Neutrophils, the most abundant leukocyte type in the human peripheral blood, have been proposed as the key elements of innate immunity in defense against infectious pathogens [90]. The NLR in peripheral blood reflects the balance between systemic inflammation and adaptive immunity. Therefore, the elevations of neutrophils and NLR in AD indicate the putative roles of the systemic inflammatory response in AD pathogenesis. Interestingly, we noticed a higher NLR in MCI than in NC, suggesting that this easily available biomedical measurement could serve as a powerful indicator for the early diagnosis of AD. Moreover, the previous studies from AD mouse models suggested that neutrophils in the peripheral blood could adhere to the brain capillaries and block cortical blood flow, extravasate into the brain parenchyma through the integrin LFA-1, and finally promote damage of the BBB [35, 36, 91]. Accordingly, we noticed an increased abundance of neutrophils in multiple brain regions at both early and late periods of AD, and a positive correlation between neutrophils abundance and AD pathological changes (e.g., tau neurofibrillary tangle and amyloid score).

Given the contribution of mitochondrial dysfunction and decreased metabolism to cognition decline and Aβ deposition [92], the specific downregulation of metabolic process and mitochondrion organization in neutrophils highlights the role of neutrophils in AD progression. Moreover, we noticed that the network modules of mitochondrion organization, cytokine-mediated signaling pathway, and leukocyte cell-cell adhesion in neutrophils were significantly enriched in AD risk or associated genes. Specifically, the expression of some proinflammatory cytokines and receptors (e.g., such as *IL1B*, *IL1R1*, *IL21R*, and *TNFAIP6*) were significantly upregulated in neutrophils in AD patients compared to controls. These cytokines could target microglia and brain endothelium cells, resulting in an increased microglial activation and altered permeability and transport of BBB [29], respectively. In addition, *CD33*, a biomarker of immature neutrophils [93], was downregulated in AD neutrophils, consistent with the elevated neutrophil degranulation in AD. Besides, the expression of oxidative phosphorylation subunit genes (e.g., *NDUFC2*, *NDUFA7*, and *NDUFV1*) was reduced in neutrophils, mirroring mitochondrial dysfunction in AD brain [94]. These results indicate the potential role of molecular changes of neutrophils in AD pathogenesis.

There is a consistency between the enrichment for neutrophils degranulation of bulk upregulated genes and the increased proportion of neutrophils. In addition, there is a significant overlap between the bulk DEGs and the intrinsic DEGs for most immune cell types, especially for neutrophils. This suggests that the bulk transcriptome alteration is driven by both cellular proportion and abundance and cellular expression changes. Despite a significant overlap, the majority of DEGs of each cell type were not detected at the bulk level, highlighting the importance to identify cellular intrinsic DEGs for the development of diagnostic biomarkers. Besides, we noticed that the DEGs showed limited overlap among different cohorts (Fig. 3C) and also limited overlap with those identified from single-cell RNA sequencing datasets (Additional file 1: Fig. S13) [38, 40, 78], which may due to the differences of sample preparation, sequencing methods, and analytic protocols, as well as the heterogeneity of the participants. However, there was a convergence in dysregulated signaling pathways, such as the dysregulations of regulation of defense response, cellular response to cytokine stimulus, and regulation of inflammatory response in the majority of cell types, suggesting that pathway and network-based markers may be more robust [95].

Despite the biological insights gained from deconvoluted cellular proportion and cellular molecular alterations, there are also some limitations. Firstly, we adjust common covariates of age and gender when performing differential expression analysis and logistic regression analysis. However, immune cell count and expressional profiling of peripheral blood are susceptible to the effects of hiding confounding factors, such as comorbidity, clinical, and lifestyle factors. Second, AD is a heterogeneous disorder with diverse pathophysiologic mechanisms. Accordingly, molecular subtypes corresponding to different combinations of multiple dysregulated pathways, such as susceptibility to tau-mediated neurodegeneration, aβ neuroinflammation, synaptic signaling, immune activity, and mitochondria organization, have been revealed by brain transcriptome data [96]. Therefore, the alterations identified in this study might reflect the average signaling of heterogeneous samples. Finally, currently there is no available single-cell RNA-seq data for the neutrophils in AD. Although the changes in cell proportions and enriched signaling pathways are reproducible across multiple independent datasets, there is a lack of comparisons of single-cell analyses on the same batch of data to illustrate the performance of the deconvolution methods.

## Conclusions

In summary, we explored the alterations of the proportions of diverse immune cell types and the changes of intrinsic expression of individual cell types in the peripheral blood from AD participants. We observed a consistent increase of the cellular proportion of neutrophils and NLR across independent cohorts in both AD and MCI participants compared to the controls. We identified cell-type-specific and shared signaling pathways based on the DEGs in the immune cell types. We then re-constructed PPI networks for the DEGs in each immune cell type and identified several modular clusters from neutrophils that are associated with AD pathogenesis, for which the molecular functions are enriched in leukocyte cell-cell activation, mitochondrion organization, and cytokine-mediated signaling pathway. Both changes of the abundance of neutrophils and the expression levels of the hub genes from the relevant modular clusters in neutrophils showed significant association with the pathological alterations in AD. This work highlights the clinical value of the cellular abundance and expression profiling of neutrophils and, in the future, integration analysis of these data in the peripheral blood will enable a monitoring of AD/MCI pathogenesis and may provide novel insights into the development of therapeutic targets for AD.

## Supplementary Information

**Additional file 1: Figure S1.** Comparison of the NLR between MCI and NC in different datasets using different computational methods. NLR, neutrophil-to-lymphocyte ratio. **Figure S2.** Correlation between immune cell proportion and cognition measurements in AD and NC individuals. Correlations were assessed using Pearson’s correlation test. MoCA-B, Montreal Cognitive Assessment Basic; MMSE, Mini-Mental State Examination. **Figure S3.** Correlation between immune cell proportion and cognition measurements in all (i.e., AD, MCI, and NC) individuals. **Figure S4.** Demographic characteristics of participants across datasets. (A) The number of individuals for each diagnosis in each dataset. (B) Age distribution of participants across diagnosis groups in each dataset. The comparison was assessed with the Wilcoxon test. (C) Gender composition of subjects across each group of each dataset. The comparison was assessed with the Fisher test. **Figure S5.** Overlap of differentially expressed genes (DEG) in AD patients identified with limma (red) and the Wilcoxon test (blue). *P*-values for hypergeometric tests of pairwise overlaps are shown at the top. **Figure S6.** Bulk DEGs in MCI compared with NC. (A) Venn diagram shows the overlap of bulk DEGs in MCI across four blood transcriptomic datasets. (B-C) The top enriched biological processes of up (B) and down (C) regulated genes in MCI compared with NC in each dataset. The point size represents the number of DE genes in the corresponding pathway. Color represents the significant level (i.e., FDR). **Figure S7.** Overlap between bulk DEGs (blue) and DEGs in each cell type (red) of AD. *P* values for hypergeometric tests of pairwise overlaps are shown at the top. **Figure S8.** Top ten hub genes with high degree centrality or clustering coefficients in Modules 2 and 3. **Figure S9.** Spearman correlation between expression levels DEGs of AD neutrophils and MRI biomarkers for AD diagnosis and progression, where only genes with significant association with at least one MRI feature were shown. **Figure S10.** Top enriched biological processes of down (A) and up (B) regulated genes in AD compared with NC. **Figure S11.** Correlation between the neutrophil abundance score and Braak tau neurofibrillary tangle staging score in the cerebellum. The association was assessed using the Pearson correlation test. **Figure S12.** Comparison of immune cell proportion in the cerebellum (A) and temporal cortex (B) between AD and NC. **Figure S13.** Overlap of cell-intrict DEGs among different dataset. Blue: our study; Red: Kuan et. al’s study; Yellow: Kuan et. al’s study.

**Additional file 2: Table S1.** Comparison of proportion of neutrophil**s**, B cells, and NLR between AD/MCI and NC with adjusting for age and sex. **Table S2.** Top enriched pathways for each PPI module cluster identified in neutrophils. **Table S3.** The complete list of enriched terms in AD compared with NC across different data cohorts. **Table S4.** The complete list of enriched terms in MCI compared with NC across different data cohorts.

## Data Availability

The ADNI expression data are available at the (Alzheimer’s Disease Neuroimaging Initiative) ADNI database (https://adni.loni.usc.edu). The ANM1 and ANM2 transcriptomic datasets are accessible at NCBI Gene Expression Omnibus with Series accession number GSE165090 and GSE63061, respectively. ZIB-AD RNA-seq data are available at Genome Sequence Archive for Human (GSA-Human) in the National Genomics Data Center (NGDC) with accession number HRA000942. The MayoRNA-seq data for brain are available from the AD Knowledge Portal (https://www.synapse.org/#!Synapse: syn5550404) upon authentication by the Consortium. The Alzmap Spatial Transcriptomics data is available in the GEO database with accession number GSE152506.

## Abbreviations

Aβ: Amyloid-β peptide
AD: Alzheimer’s disease
ADNI: Alzheimer’s Disease Neuroimaging Initiative
CBE: Cerebellum
CX: Cortex
DEG: Differentially expressed gene
GWAS: Genome-wide association studies
HP: Hippocampus
MCI: Mild cognitive impairment
NFT: Neurofibrillary tangles
NINCDS-ADRDA: National Institute of Neurological and Communicative Disorders and Stroke-Alzheimer's Disease and Related Disorders Association
NLR: Neutrophil-to-lymphocyte ratio
PCA: Principal component analysis
PPI: Protein-protein interaction
RMA: Robust Multichip Analysis
TCX: Temporal cortex
ZIB: Zhangjiang international Brain Biobank
