# Supplementary material for: Cellular transcriptional alterations of peripheral blood in Alzheimer’s disease
Source: BMC Med. 2022 Aug 29;20:266. doi: 10.1186/s12916-022-02472-4 (PMC9422129; doi:10.1186/s12916-022-02472-4)
Supplement: Supplementary file 1 — Additional file 1: Figure S1. Comparison of the NLR between MCI and NC in different datasets using different computational methods. NLR, neutrophil-to-lymphocyte ratio. Figure S2. Correlation between immune cell proportion and cognition measurements in AD and NC individuals. Correlations were assessed using Pearson’s correlation test. MoCA-B, Montreal Cognitive Assessment Basic; MMSE, Mini-Mental State Examination. Figure S3. Correlation between immune cell proportion and cognition measurements in all (i.e., AD, MCI, and NC) individuals. Figure S4. Demographic characteristics of participants across datasets. (A) The number of individuals for each diagnosis in each dataset. (B) Age distribution of participants across diagnosis groups in each dataset. The comparison was assessed with the Wilcoxon test. (C) Gender composition of subjects across each group of each dataset. The comparison was assessed with the Fisher test. Figure S5. Overlap of differentially expressed genes (DEG) in AD patients identified with limma (red) and the Wilcoxon test (blue). P-values for hypergeometric tests of pairwise overlaps are shown at the top. Figure S6. Bulk DEGs in MCI compared with NC. (A) Venn diagram shows the overlap of bulk DEGs in MCI across four blood transcriptomic datasets. (B-C) The top enriched biological processes of up (B) and down (C) regulated genes in MCI compared with NC in each dataset. The point size represents the number of DE genes in the corresponding pathway. Color represents the significant level (i.e., FDR). Figure S7. Overlap between bulk DEGs (blue) and DEGs in each cell type (red) of AD. P values for hypergeometric tests of pairwise overlaps are shown at the top. Figure S8. Top ten hub genes with high degree centrality or clustering coefficients in Modules 2 and 3. Figure S9. Spearman correlation between expression levels DEGs of AD neutrophils and MRI biomarkers for AD diagnosis and progression, where only genes with significant association with at least o [file 12916_2022_2472_MOESM1_ESM.docx]

**Additional File 1 for**

**Cellular transcriptional alterations of peripheral blood in Alzheimer’s disease**

Liting Song^1^, Yucheng T. Yang^1,2,3^, Qihao Guo^4^, the ZIB Consortium, Xing-Ming Zhao^1,2,3,5,*^

^1^ Institute of Science and Technology for Brain-Inspired Intelligence, Fudan University, Shanghai 200433, China

^2^ MOE Key Laboratory of Computational Neuroscience and Brain-Inspired Intelligence, and MOE Frontiers Center for Brain Science, Fudan University, Shanghai 200433, China.

^3^ Zhangjiang Fudan International Innovation Center, Shanghai 200433, China

^4^ Department of Gerontology, Shanghai Jiao Tong University Affiliated Sixth People’s Hospital, Shanghai 200233, China

^5^ International Human Phenome Institutes (Shanghai), 200433, China

^*^ To whom correspondence should be addressed. Email: Xing-Ming Zhao: [xmzhao@fudan.edu.cn](mailto:xmzhao@fudan.edu.cn).

Figures S1-S13

**Figure S1. Comparison of the NLR between MCI and NC in different datasets using different computational methods.** NLR, neutrophil-to-lymphocyte ratio.

**Figure S2. Correlation between immune cell proportion and cognition measurements in AD and NC individuals.** Correlations were assessed using Pearson’s correlation test. MoCA-B, Montreal Cognitive Assessment Basic; MMSE, Mini-Mental State Examination.

**Figure S3. Correlation between immune cell proportion and cognition measurements in all (i.e., AD, MCI, and NC) individuals.**

**Figure S4. Demographic characteristics of participants across datasets.** (A) The number of individuals for each diagnosis in each dataset. (B) Age distribution of participants across diagnosis groups in each dataset. The comparison was assessed with the Wilcox test. ns, not significant; ., *P* < 0.1; *, *P* ≤ 0.05; **, *P* ≤ 0.01; ***, *P* ≤ 0.001. (C) Gender composition of subjects across each group of each dataset. The comparison was assessed with the Fisher test.

**
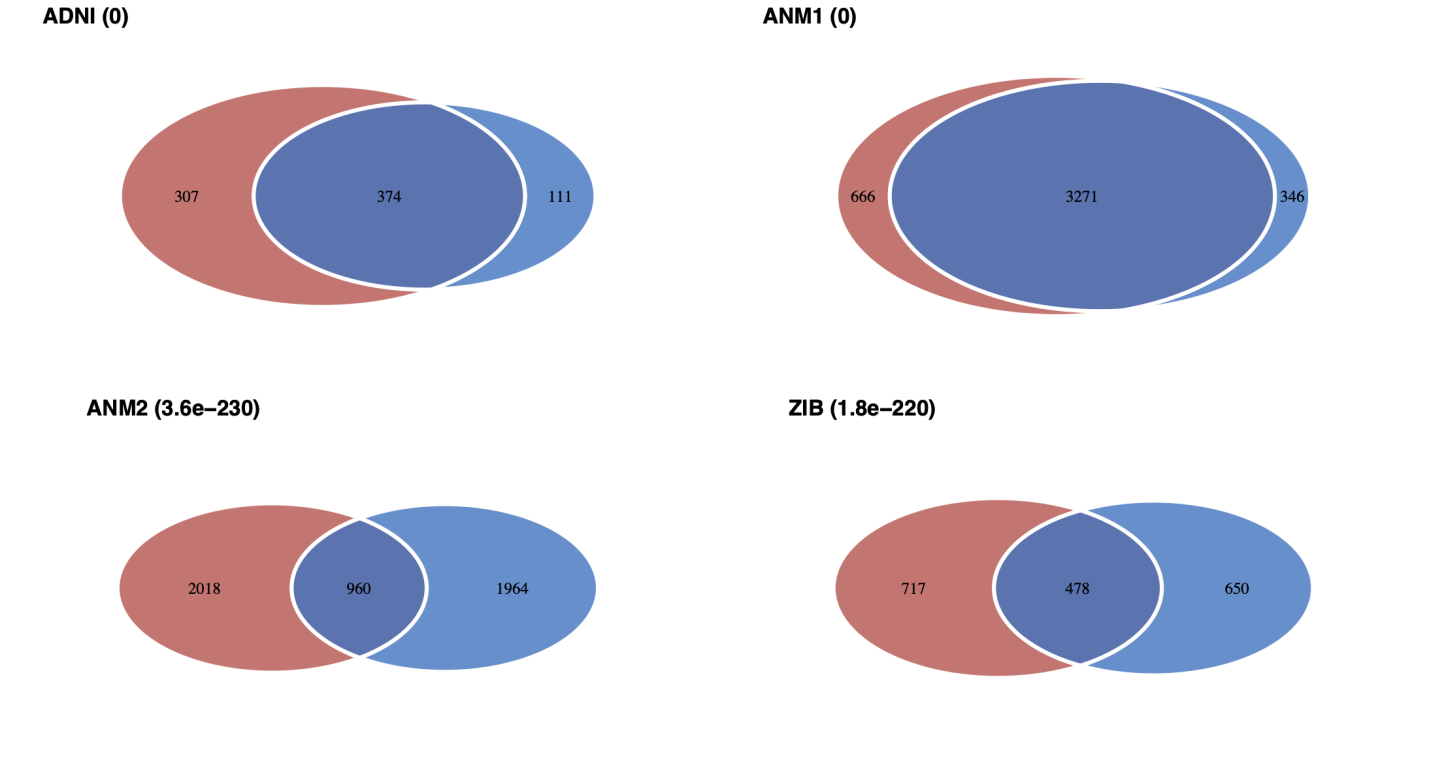
**

**Figure S5. Overlap of differentially expressed genes (DEG) in AD patients identified with limma (red) and the Wilcoxon test (blue).** P values for hypergeometric tests of pairwise overlaps are shown at the top.


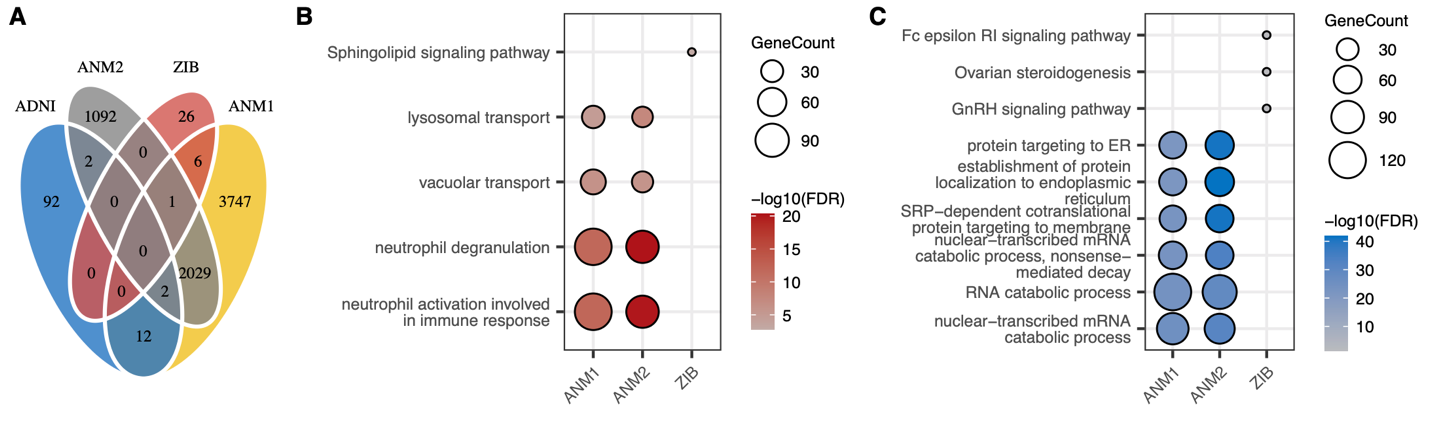


**Figure S6. Bulk DEGs in MCI compared with NC.** (A) Venn diagram shows the overlap of bulk DEGs in MCI across four blood transcriptomic datasets. (B-C) The top enriched biological processes of up (B) and down (C) regulated genes in MCI compared with NC in each dataset. The point size represents the number of DE genes in the corresponding pathway. Color represents the significant level (i.e., FDR).


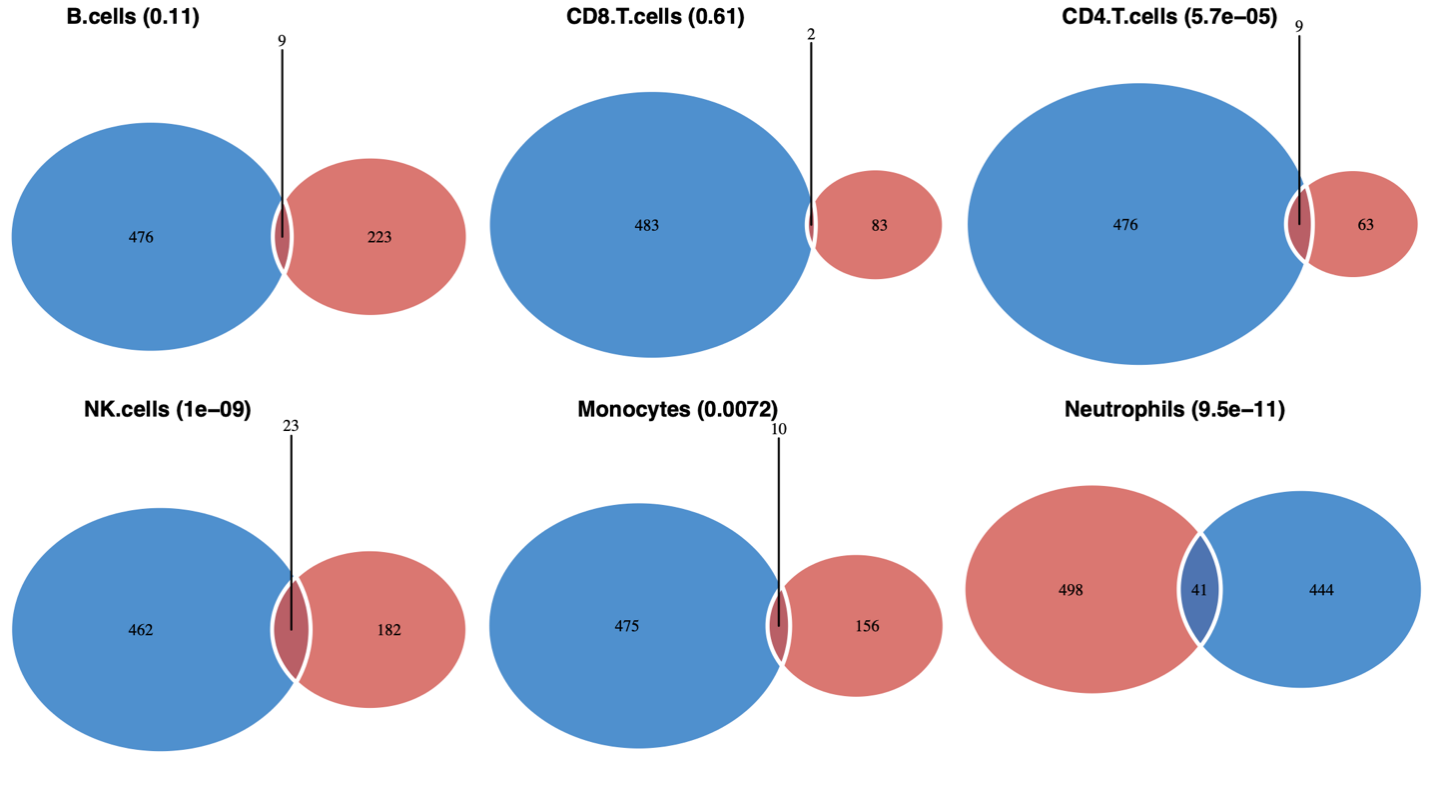


**Figure S7. Overlap between bulk DEGs (blue) and DEGs in each cell type (red) of AD.** P values for hypergeometric tests of pairwise overlaps are shown at the top.

**Figure S8**. **Top ten hub genes with high degree centrality or clustering coefficients in Modules 2 and 3.**

**Figure S9**. **Spearman correlation between expression levels DEGs of AD neutrophils and MRI biomarkers for AD diagnosis and progression, where only genes with significant association with at least one MRI feature were shown.**

**Figure S10. Top enriched biological processes of down (A) and up (B) regulated genes in AD compared with NC.**

**Figure S11. Correlation between the neutrophil abundance score and Braak tau neurofibrillary tangle staging score in the cerebellum.** The association was assessed using the Pearson correlation test.

**Figure S12. Comparison of immune cell proportion in the cerebellum (A) and temporal cortex (B) between AD and NC.**


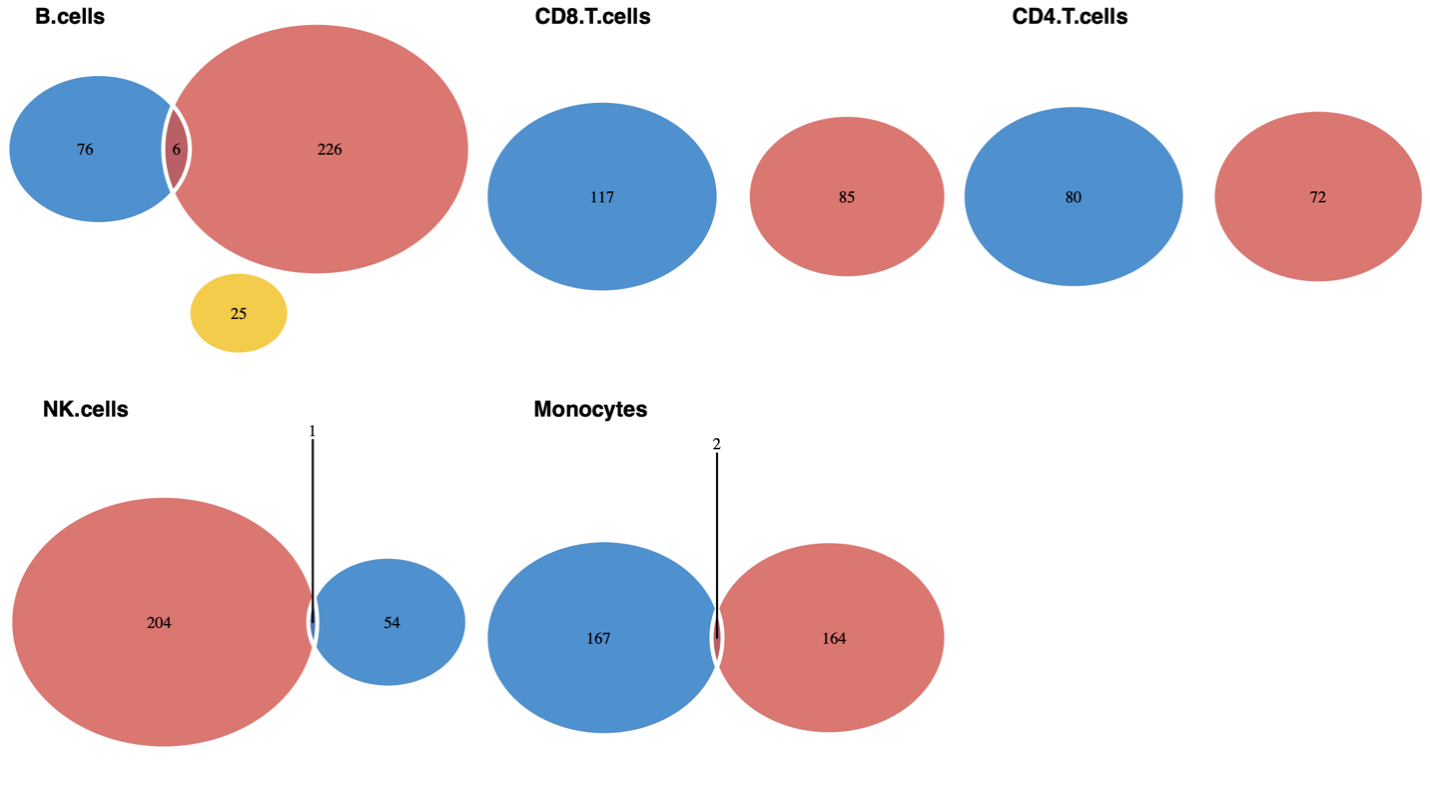


**Figure S13. Overlap of cell-intrict DEGs among different dataset.** Blue: our study; Red: Kuan et. al’s study; Yellow: Kuan et. al’s study.
